# From Neurophobia to Neuroanxiety: An Opportune Review of Its Definition and Impact on Neurology Educators and Learners

**DOI:** 10.1212/NE9.0000000000200247

**Published:** 2025-08-26

**Authors:** Youjiang Tan, Shawn Z.Z. Lin, Zhibin Tan

**Affiliations:** Department of Neurology, National Neuroscience Institute, Singapore General Hospital Campus.

In 1994, Jozefowicz^1^ first coined the term “neurophobia” to describe a “fear of neural sciences and clinical neurology resulting from students' inability to apply their knowledge of basic sciences to clinical scenarios,” which has since been widely accepted as the main definition of this phenomenon. The use of “phobia” proved problematic because it narrowly confines these sentiments to fear, a highly intense and irrational emotion that is disproportionate to the actual threat, ignoring milder emotional responses where early intervention may be more effective.^2^ Thirty years on, emerging research has brought forth a range of new perspectives—suggesting that the emotional responses associated with neurophobia may be more diverse, rational, and contextually justified than the “phobia” implies, rendering it timely to revisit how the phenomenon is being understood and defined.^3-5^

Our systematic search on PubMed and Google Scholar for articles on neurophobia published between 1994 and 2024 (n = 119) uncovered a remarkable prominence of “neurology” and “fear” when defining neurophobia, followed by “neuroscience/neurosciences/neural sciences,” “students,” and “knowledge”^4,5^ (see eReferences e1–e118). To elucidate evolving trends when defining neurophobia, we then compared articles published in the past 5 years (n = 77) against earlier publications (n = 45)e119. While we found similar frequencies in the use of “fear” and “students,” we also observed an increase in the use of “neuroscience/neural science” instead of “neurology” in more recent literature, along with alternative descriptors of negative sentiments such as anxiety, apprehension, and aversion, reflecting evolving perceptions of neurophobia over time.

To explore the evolving and prevailing trends of how neurophobia is/was perceived, we surveyed our health care community between November 2024 and March 2025, using a questionnaire (eAppendix 1) that incorporated key components from existing neurophobia screening tools.^e2,e7^ Multiple responses were permitted where applicable. Doctors and neuroscience nurses from the Departments of Medicine and Neurology at Singapore General Hospital, and medical students on their neurology rotations, were invited to participate (IRB 2024–4646) through mass messaging. In this study, house officers (postgraduate year 1) and residents (postgraduate year 2 and above) were classified as doctors-in-training. A total of 103 respondents completed the survey (approximately 35% response rate), comprising 5 neuroscience nurses, 25 medical students, 36 doctors-in-training, 18 neuroscience specialists, and 19 non-neuroscience specialists. Our findings revealed that 54% disagreed with Jozefowicz's definition because its focus on fear inappropriately reduces a complex and highly nuanced phenomenon to that of a singular, strong, and intense emotional response, thereby failing to capture milder but still meaningful sentiments. Less than half (41%) agreed that neurophobia stems primarily from difficulties when translating basic science to clinical practice, with some pointing to neurosciences' inherent complexity and breadth as additional key contributors. Others further described a bidirectional relationship between the two—where neurophobia is both the cause and effect of impaired clinical application of basic neuroscience knowledge. Against this backdrop of definitional deficiencies, we then asked our respondents about how neurophobia should be defined. Most opined that the negative emotions associated with this phenomenon, its impact, and the scientific domains it involves were the key elements when describing and defining the phenomenon. Most of them identified anxiety (81%)—rather than fear (42%)—as the emotion most associated with neurophobia, which in turn impairs one's ability to assess (70%) and manage (69%) neurologic patients, leading to the avoidance of clinical and emergency situations (63%). Furthermore, 95% felt that neurophobia involved multiple neuroscience domains rather than neurology alone.

Anxiety—unlike fear—is less specific, longer lasting, and anticipatory.^e120^ Its tendency to persist further underscores the need for ongoing surveillance and sustained mitigating strategies beyond the confines of medical schools—such as curriculum reform, team-based learning, mentorship, and continuous education. Its anticipatory nature may drive avoidant behaviors before emotions intensify while its persistence helps explain why such negative sentiments extend beyond students, permeating and persisting among primary care physicians and non-neuroscience specialists as well.^4,e2,e58,e71^ Conversely, the same anticipatory character also provides a window of opportunity to intervene before triggering situations (e.g., challenging tutorials or complex clinical cases) occur, through measures such as supervision, peer support, or targeted resources.^4^^,e6,e56^ Finally, the presence of avoidant behaviors toward neuroscience-related clinical scenarios may also help educators identify affected individuals early, possibly through longitudinal observation and feedback from their peers and supervisors. However, the optimal methods and frequency for collecting such feedback remain insufficiently explored and warrant further investigation.

Another survey finding was that most respondents viewed early exposure experiences (e.g., learning complicated neurologic concepts) and negative clinical encounters (e.g., encountering patients with complex neurologic conditions) as the 2 situations when learners are at highest risk of developing anxiety toward neuroscience. Recognizing these high-risk situations can present educators with preemptive opportunities to intervene before their occurrence, by using strategies such as the reduction of extraneous cognitive load (chunking, focusing on key concepts) and optimizing germane cognitive load (schema development, problem-based learning) in the design of their lesson plans.^e121–123^ Similarly, learners can be front-loaded with the prerequisite knowledge and skills, or be provided with relevant learning materials, before participating in neuroscience-related encounters.

Despite being limited by a low response rate and the under-representation of several key groups (e.g., pediatricians, primary care physicians, neurosurgeons, and nurses) whose work frequently involves patients with neuroscience-related issues and may thus hold valuable perspectives on this topic, our findings nevertheless support a paradigm shift from “neurophobia” to “neuroanxiety”—which we defined as a spectrum of anticipatory negative sentiments toward neuroscience-related clinical/nonclinical encounters that can impair one's ability to assess and manage such patients, or lead to avoidance of such scenarios—as it better captures the emotional and behavioral responses of affected individuals to neuroscience-related issues. This shift supports and underscores the need for long-lasting mitigating strategies, while providing a framework for earlier identification of neuroanxious individuals, the implementation of effective interventions, and future research into the phenomenon.
